# Engineering *Thermothelomyces heterothallica* for cellobionate production

**DOI:** 10.1186/s40643-026-01126-0

**Published:** 2026-09-03

**Authors:** Bakht Zada, Hamidreza Shapouri, Jiajie Wang, Sumit Sharma, Takao Kasuga, Noelia Valbuena, Ronen Tchelet, Mark Emalfarb, Zhiliang Fan

**Affiliations:** 1 Department of Biological and Agricultural Engineering, One Shields Avenue, University of California, Davis, CA 95616 USA; 2 Department of Plant Pathology, One Shields Avenue, University of California, Davis, CA 95616 USA; 3 United States Department of Agriculture, Agricultural Research Service, University of California, Davis, CA 95616 USA; 4 Dyadic International, Inc., 1044 North U.S. Highway One, Suite 201, Jupiter, FL 33477 USA

**Keywords:** Cellobionate, Cellulose, *Thermothelomyces heterothallica*, β-glucosidase, Cellobiose phosphorylase

## Abstract

In this study, a thermophilic cellulolytic filamentous fungus *Thermothelomyces heterothallica* was engineered for direct production of cellobionic acid (CBA) from alkali-pretreated wheat straw without the addition of external cellulases. The CBA bioproduction pathway was established through sequential disruption of genes involved in cellobiose and CBA catabolism, including eight β-glucosidases, one cellobiose/cellobionate phosphorylase, and two putative cellobionate transporters using a ribonucleoprotein (RNP)-based CRISPR-Cas9 system. The resulting *T. heterothallica* strain, TH11, was able to produce cellobionate from cellobiose and pretreated wheat straw at a high yield. Further optimization of fermentation pH using 50 mM citrate buffer (pH 6.0) increased the final cellobionate concentration. Fermentation temperature optimization showed that 43 °C accelerated substrate conversion without significantly affecting final titers. The engineered strain, TH11 produces 64 mM of cellobionate in 5 days. The cellulose conversion was about 88%, and the yield of the cellobionate from the consumed cellulose was about 96%. The new strain provides a promising platform for direct bioconversion of lignocellulosic biomass into value-added aldonic acids without any enzyme addition.

**Graphical abstract:**

**Figure:**
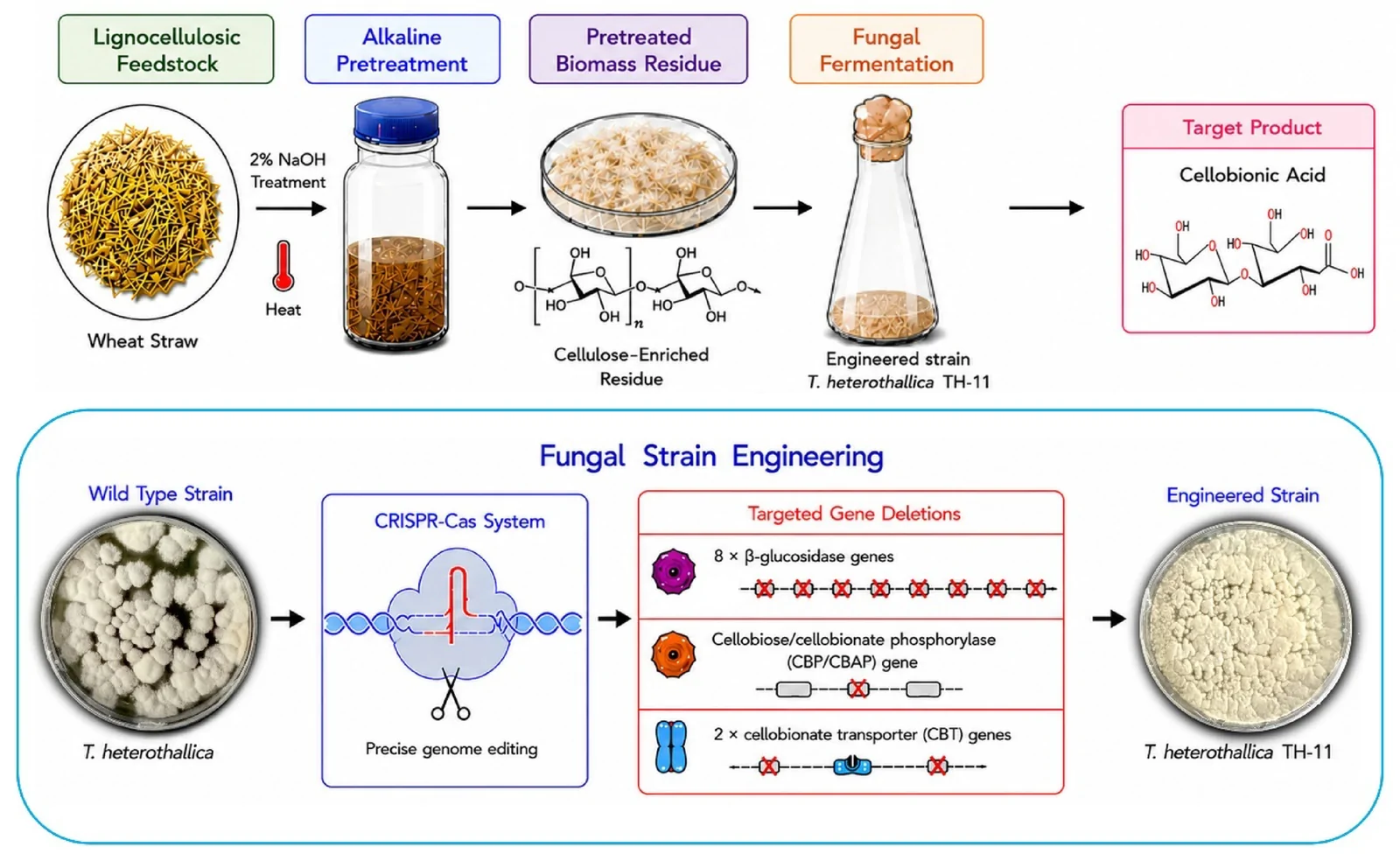

**Supplementary Information:**

The online version contains supplementary material available at https://doi.org/10.1186/s40643-026-01126-0.

## Introduction

The microbial production of organic acids offers a promising alternative to conventional chemical synthesis. Both native and genetically engineered microorganisms have been extensively used to produce a wide range of organic acids. Among these, cellobionic acid (CBA) is a value-added sugar acid derived from the oxidation of cellobiose, which has gained increasing attention because of its broad application in the food, pharmaceutical, and chemical industries (Bieringer et al. 2023). CBA, or its salt form cellobionate, can be produced from cellobiose through either biological or chemical methods (Bieringer et al. 2024; Liu et al. 2024; Morejón et al. 2023; Oh and Eom 2022; Oh et al. 2022). The high cost of cellobiose remains a major contributor to overall processing expenses. To address this issue, Yoo et al. (2022) evaluated wastepaper as a low-cost feedstock and obtained 23 g/L (67.2 mM) cellobiose through cellulase-mediated hydrolysis, followed by cellobionate production using recombinant *Pseudomonas taetrolens.* However, the dependence on exogenous cellulases and the relatively low overall product yields continue to pose significant challenges for industrial-scale application (Yoo et al. 2022).

**Fig. 1:**
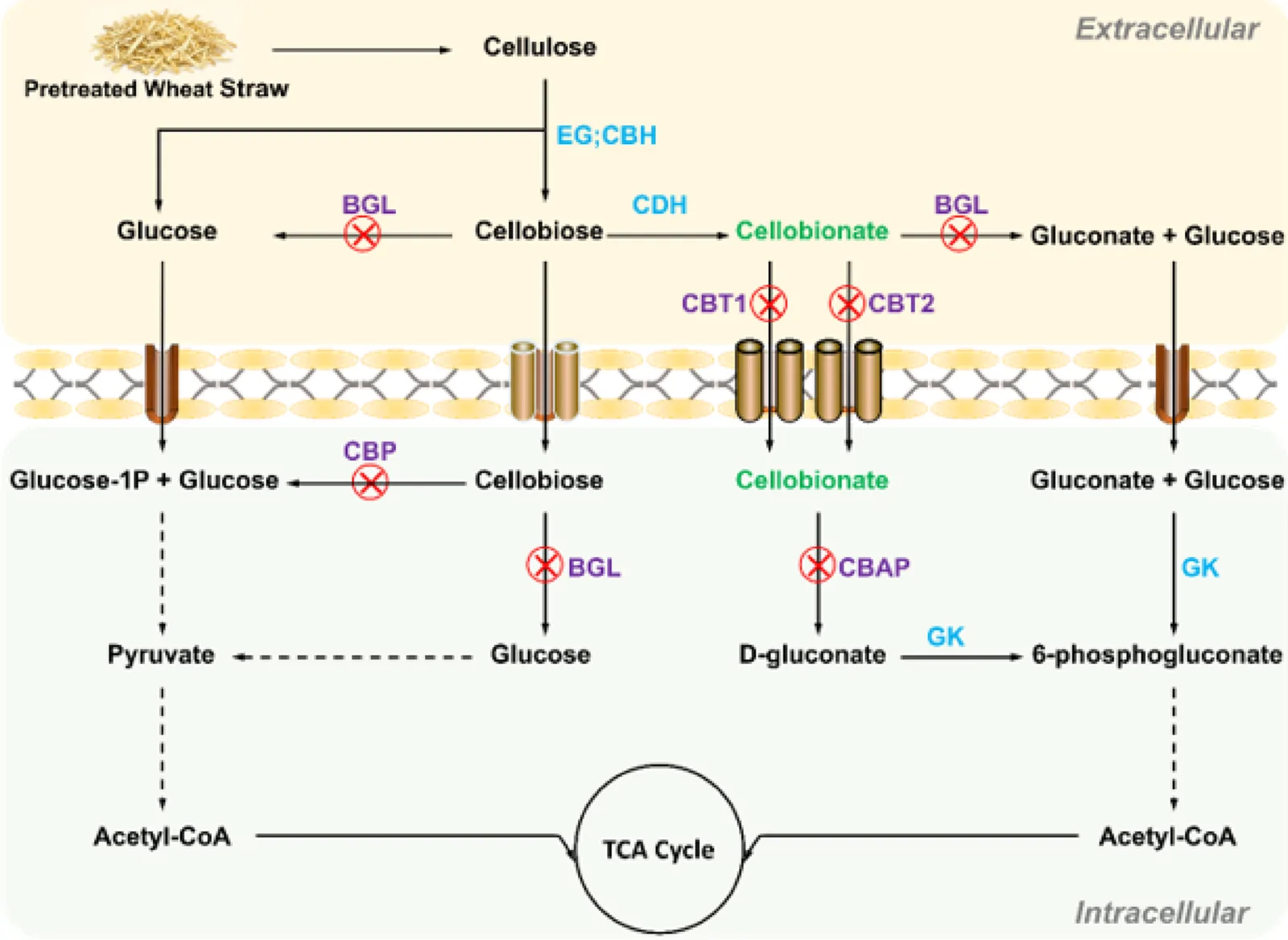
Schematic description of the strain engineering strategy for redirecting cellobiose and cellobionate flux. EG: endoglucanase, CBH: cellobiohydrolase, CBP: cellobiose phosphorylase; Glucose−1P: Glucose−1-phosphate, TCA: Tricarboxylic Acid Cycle

The production of cellobionate directly from lignocellulosic biomass is particularly attractive because it enables the valorization of abundant, renewable, and low-cost feedstock. In our prior studies, we adopted a metabolic engineering strategy to redirect cellulose toward cellobionate production by an engineered fungal strain. A cellulolytic strain, *Neurospor*a *crassa,* that could secrete a complete cellulase system for the hydrolysis of cellulose to cellobiose was first employed (Eberhart et al. 1977). As shown in Fig. 1, multiple genes encoding intracellular and extracellular β-glucosidases were deleted to promote cellobiose accumulation and prevent its further hydrolysis to glucose in *N. crassa* (Wu et al. 2013). In parallel, the endogenous cellobionate phosphorylase gene (*ndvB*), responsible for the utilization of both cellobiose and cellobionate, was removed to block downstream catabolism. To further enhance cellulose deconstruction and relieve carbon catabolite repression, two transcription factor genes, *cre-1* (NCU08807) and *ace1* (NCU09333), which encode a carbon catabolite repressor protein and a cellulase repressor, respectively, were deleted, resulting in increased cellulase production and improved carbon utilization, resulting in the strain F5Δ*ace-1*Δ*cre-1*Δ*ndvB* (Hildebrand et al. 2015). However, cellobionate production was limited by inefficient electron transfer during the oxidation process. To address this limitation, Lin et al. heterologously expressed a *Botrytis aclada* laccase gene in *N. crassa* F5Δ*ace-1*Δ*cre-1*Δ*ndvB* under the control of a copper-inducible promoter, generating the strain *N. crassa* HL10 (Lin et al. 2017). This engineered strain was capable of producing cellobionate at a titer of 57 mM from 2% NaOH-pretreated wheat straw in eight days without any enzyme addition (Wang et al. 2024). The overall yield was 90%.

Although *N. crassa* serves as an excellent model organism for developing and validating genetic engineering strategies, its cellulase production capacity is substantially lower than that of industrial cellulase-producing fungi. To construct a commercial-ready strain, we applied the same engineering strategy to the industrialized thermophilic strain *Thermothelomyces heterothallica*, which was previously classified as *Chrysosporium lucknowense* and *Myceliophthora thermophila* (Visser et al. 2011)*.* As a thermophilic strain, it offers significant process benefits. It is capable of robust growth at elevated temperatures (40–55 °C), which reduces contamination risk and accelerates reaction kinetics (da Rosa-Garzon et al. 2022; Visser et al. 2011). Moreover, this industrialized organism has an exceptional capacity for native and heterologous protein expression and secretion, enabling high-level production of extracellular protein of more than 100 g/L (Keresztes et al. 2022; Singh 2016; Visser et al. 2011). These characteristics make *T. heterothallica* particularly attractive for industrial CBA production, where high enzyme secretion, robust growth, and operation at elevated temperatures could reduce process costs and improve productivity. Importantly, the use of an industrially established fungal host also provides a more practical foundation for scaling CBA production. Starting from *T. heterothallica*, we constructed the CBA production strain by knocking out eleven genes, including one cellobiose phosphorylase (CBP), eight β-glucosidases (BGLs), and two cellobionate transport proteins (CBTs) (Fig. 1).

This study reports the construction and evaluation of mutant strains for cellobionate production and optimization of process conditions to improve cellobionate yields.

## Materials and methods

### Strains and culture conditions

*T. heterothallica* TH001 (referred to as TH0 in this study), a strain developed by Dyadic International, served as the starting strain for genetic engineering. All *T. heterothallica* strains were cultured on isotonic/isoosmotic minimal medium (IMM) agar plates containing 1.5% Agar Noble, 670 mM sucrose, 1% glucose, 70 mM NaNO_3_, 7 mM KCl, 11 mM KH_2_PO_4_, 2 mM MgSO_4_, 174 μM EDTA, 76 μM ZnSO_4_·7H_2_O, 178 μM H_3_BO_3_, 25 μM MnSO_4_·H_2_O, 18 μM FeSO_4_·7H_2_O, 7.1 μM CoCl_2_·6H_2_O, 6.4 μM CuSO_4_·5H_2_O, and 6.2 μM Na_2_MoO_4_·2H_2_O. Plates were incubated at 35 °C for 5–7 days. For liquid culture, the rich medium with yeast extract (RMYE) was used, consisting of 0.6% yeast extract, 1% glucose, 35 mM (NH_4_)_2_SO_4_, 7 mM KCl, 55 mM KH_2_PO_4_, 2 mM MgSO_4_, 174 μM EDTA, 76 μM ZnSO_4_·7H_2_O, 178 μM H_3_BO_3_, 25 μM MnSO_4_·H_2_O, 18 μM FeSO_4_·7H_2_O, 7.1 μM CoCl_2_·6H_2_O, 6.4 μM CuSO_4_·5H_2_O, and 6.2 μM Na_2_MoO_4_·2H_2_O. Cultures were grown at 35 °C with shaking at 230 rpm for 22 h. Antibiotics were added as needed for transformant selection, and Penicillin/Streptomycin solution (50 U/mL penicillin and 0.05 mg/mL streptomycin) was added to prevent bacterial contamination.

For DNA manipulation, *Escherichia coli* DH5α was used. This strain was cultivated at 37 °C with shaking at 250 rpm in LB (Luria–Bertani) medium containing 10 g tryptone, 5 g yeast extract, and 10 g sodium chloride per liter, provided with 100 µg/mL ampicillin or 50 µg/mL kanamycin for plasmid selection.

### RNP-based CRISPR Cas9 system for strain engineering

A Ribonucleoprotein (RNP)-based CRISPR-Cas9 genome editing system was employed for targeted gene knockout in *T. heterothallica*. The RNP complex consisted of the Cas9 endonuclease, CRISPR-Cas9 tracrRNA, and CRISPR-Cas9 crRNA, all obtained from Integrated DNA Technologies, Inc. (IDT) (Coralville, IA 52241, USA). Strain engineering was carried out through four sequential rounds of CRISPR-Cas9-mediated gene disruption. The parent strain *T. heterothallica* TH0 was used as the starting strain for all genetic manipulations. In the first round, a hygromycin resistance cassette was used to simultaneously knock out the cellobiose/cellobionate phosphorylase gene (*cbp*/*cbap*) and two β-glucosidase genes (*bgl1* and *bgl2*). The second round employed a phosphinothricin (*bar*) resistance cassette to knock out *bgl3*, *bgl4*, and *bgl5*, followed by knocking out *bgl6*, *bgl7*, and *bgl8* using a neomycin resistance cassette in the third round. In the final round, we used a zeocin resistance cassette to knock out the two putative cellobionate transporter genes (*cbt1* and *cbt2*), generating the final strain, TH11. For each target gene, two CRISPR-Cas9 crRNAs were designed using the IDT online design tools. All PCR primers and CRISPR-Cas9 crRNAs used in this study are detailed in the Supplementary file, Tables S1 and S2, respectively. The list of targeted genes is detailed in the Supplementary file, Table S3.

The antibiotic cassettes were prepared by either PCR amplification of existing plasmids or commercially synthesized by Azenta Life Sciences (Santa Clara, CA, USA). The detailed sequences are listed in Table S3. To perform simultaneous gene knockout, the donor antibiotic cassette (hygromycin, bar, neomycin, or zeomycin), Cas9 endonuclease, CRISPR-Cas9 tracrRNA, and CRISPR-Cas9 crRNAs were mixed and co-transformed into the *T. heterothallica* using a PEG-mediated protoplast transformation protocol. Briefly, strains were cultivated in 50 mL RMYE medium at 35 °C and 230 rpm for 22 h. Mycelia were harvested by centrifugation (4000 rpm, 15 min), washed with 0.6 M NaCl–0.27 M CaCl_2_, and divided into aliquots containing ≤ 3 g wet biomass. The cell wall-degrading enzyme, yatalase (Takara Bio Inc., Shiga, Japan), was used at 30 mg g^−1^ wet biomass. The enzyme was dissolved in 0.6 M NaCl–0.27 M CaCl_2_, filter-sterilized, and added at 5 mL g^−1^ biomass. Suspensions were incubated at 30 °C and 70 rpm for 2–3 h until protoplast formation was observed microscopically. Protoplasts were filtered through sterile Miracloth, washed twice with cold STC buffer solution (1.2 M sorbitol, 50 mM CaCl_2_·2H_2_O, 35 mM NaCl, 10 mM Tris), and recovered by centrifugation at 1500 × g for 10 min at 4 °C. The final protoplast suspension was adjusted to approximately 3 × 10^8^ protoplasts mL^−1^ in STC buffer and maintained on ice. 25 μL of RNP-mix (540 nM per each gRNA), which consisted of 1× Cas9 buffer, CRISPR-Cas9 crRNA:tracrRNA (1 μM), and Cas9 endonuclease, was mixed with 180 μL of protoplast suspension and 7.5 μg donor DNA, followed by incubation at room temperature for 25 min. Subsequently, 2 mL of 60% PEG 4000 solution (60% PEG 4000, 50 mM CaCl_2_·2H_2_O, 35 mM NaCl, 10 mM Tris) was added dropwise, and samples were incubated for an additional 20 min. STC buffer was added to a final volume of ~11 mL, and protoplasts were collected by centrifugation (1500 × g, 10 min), resuspended in 5 mL STC buffer, and incubated overnight at 30 °C and 70 rpm. The following day, cells were pelleted, resuspended in STC buffer, and plated onto selective agar. To ensure the isolation of single colonies, 100 μL of the transformation mixture was diluted with 300 μL STC buffer before plating. Plates were incubated at 35 °C (protoplast side facing up), inverted after 24 h, and cultured until transformant colonies appeared (typically 7–10 days). Putative transformants were selected on plates containing the appropriate antibiotics: hygromycin B (100 μg mL^−1^), phosphinothricin (50 μg mL^−1^), G418 (200 μg mL^−1^), or zeocin (100 μg mL^−1^). Correct integration and targeted gene disruption were verified by PCR and confirmed subsequent sequencing.

### Characterization of glucose, cellobiose, cellobionate, and pretreated wheat straw utilization by the parental and mutant strains

The parental strain and the mutants were cultivated to evaluate substrate consumption and cellobionate production in shake flasks. All experiments were carried out in triplicate. Briefly. 0.5 mL of fresh mycelium harvested from an agar plate was inoculated into 50 mL of sterile RM-YE in a 250 mL flask and incubated at 35 °C, 230 rpm, for 18–22 h. Subsequently, 1 mL of the resulting seed culture was inoculated into 50 mL of fermentation medium containing 1× Vogel’s salts, 20 g/L glucose, 20 g/L cellobiose, 10 g/L cellobionate, or 2% alkali (NaOH) pretreated wheat straw, at a solid loading equivalent to 20 g/L cellulose in a 250 mL flask. In the medium containing wheat straw, a small amount of glucose (3 g/L) was supplied to facilitate the growth of the initial inoculum. In the medium containing cellobiose or cellobionate, glucose was supplied to 10 g/L. Unless otherwise specified, shake-flask fermentations were carried out at 40 °C and 230 rpm for 5 days. For temperature-optimization experiments, cultures were evaluated at 37, 40, 43, and 46 °C. Samples were collected at different time intervals for sugars and cellobionate analysis.

### Sample analysis

To quantify sugars and cellobionate concentrations, 1 mL of fermented broth was collected and immediately heated in a boiling water bath for about 10 min to deactivate enzyme activity. The samples were subsequently centrifuged to remove solids, and the supernatant was mixed with Tris–HCl (pH 8.0) to neutralize the samples before analysis. The samples were analyzed using a HPLC system equipped with a refractive index (RI) detector. Separation was achieved using a CarboSep CHO-87C (Concise Separations, USA) column at 80 °C. 3 mM CaCl_2_ was used as the mobile phase at a flow rate of 0.6 mL/min.

### Enzyme activity assays

Cellobiohydrolase (CBH) activity was assayed using p-nitrophenyl-β-D-lactoside (pNPL) as substrate following a modified protocol (Lin et al. 2017). The reaction mixture, containing 80 μL of 1 mg/mL pNPL and 80 μL of diluted culture supernatant, was incubated at 37 °C for 30 min. The reaction was terminated by adding 80 μL of 150 mM NaOH, and the released p-nitrophenol was quantified by measuring absorbance at 405 nm. One unit of CBH activity was defined as the amount of enzyme required to release 1 μmol of p-nitrophenol per minute.

Cellobiose dehydrogenase (CDH) activity was measured based on 2,6-dichlorophenolindophenol (DCPIP) reduction at 515 nm in 96-well plates at 30 °C using cellobiose as the substrate (Baminger et al. 1999; Hildebrand et al. 2015). The assay mixture contained 0.25 mM DCPIP, 75 mM sodium acetate buffer, 3.75 mM cellobiose, and 5 mM sodium fluoride. One unit of CDH activity was defined as the amount of enzyme required to reduce 1 μmol of DCPIP per minute.

### Yield and conversion calculations

Cellulose conversion was quantified as the percentage of the initial cellulose that was consumed during the process. Similarly, xylan conversion was determined as the percentage of the initial xylan utilized. Cellobionate yield was calculated by dividing the molar amount of cellobionate produced by the molar amount of cellulose consumed, with cellulose moles estimated using a molecular weight of 324 g mol^−1^.

### Statistical analysis

All fermentation experiments were performed in triplicate. The analyzed data are presented as the mean ± standard deviation (SD) of three replicates. Statistical analysis was performed using Microsoft Excel.

## Results and discussion

### Composition of the alkali-pretreated wheat straw

Wheat straw, ground to a particle size of ≤ 1 mm, was used as the lignocellulosic feedstock for this study. Before fermentation, the wheat straw was pretreated with 2% sodium hydroxide to enhance the cellulose accessibility to the enzymes secreted by the strain (Wang et al. 2024). The glucan, xylan, and lignin contents of the pretreated biomass were determined following the standard procedure outlined in NREL/TP-510–42618 (Sluiter et al. 2008). Batch 1 of the alkali-pretreated biomass contained 52.3 ± 0.4% glucan, 25.3 ± 0.2% xylan, and 13.4 ± 2.0% lignin, whereas Batch 2 contained 55.4 ± 0.4% glucan, 26.2 ± 0.2% xylan, and 12.1 ± 1.1% lignin. The temperature-effect experiments described below were conducted using wheat straw from Batch 2, while all other experiments utilized wheat straw produced in Batch 1.

### Construction of mutant strains and glucose utilization by parental and mutant strains

In order to redirect cellulose-derived carbon toward cellobionate production, genes involved in cellobiose and cellobionate catabolism, and cellobionate transportation were sequentially knocked out. These targets included the cellobionate phosphorylase gene (*cbp*), β-glucosidase genes (*bgls*), and cellobionate transporter genes (*cbt*). In *T. heterothallica* TH0, one *cbp*, two *cbt*, and eight putative *bgl* genes (*bgl1*–*bgl8*) were identified based on genome annotation. The β-glucosidase genes were designated *bgl1–bgl8* based on their relative expression levels determined from transcriptomic analysis, with the numbering assigned in descending order of expression (i.e., *bgl1* exhibited the highest expression level) (Li et al. 2019; Qin et al. 2022)). Protein subcellular localization was predicted using SignalP (Armenteros et al. 2019). The target genes, and their gene ID, locus tag, and gene product location are summarized in Table S3. In the first round of strain engineering, *cbp*, *bgl1*, and *bgl2*, encoding the most highly expressed intracellular and extracellular β-glucosidases, were knocked out simultaneously to generate strain TH3. In the second and third rounds, *bgl3* to *bgl5* and *bgl6* to *bgl8* were sequentially knocked out, yielding strains TH6 and TH9, respectively. Finally, two putative cellobionate transporter genes *cbt1* and *cbt2* were knocked out in TH9 to generate the final production strain, TH11. The strains and their genotypes were listed in Table 1.

**Table 1:** List of *T. heterothallica* strains used in this study

Strain	Genotype	References
TH0	*T. heterothallica fDNL_0001*	Dyadic Int
TH3	*T. heterothallica*, *Δbgl1*, *Δbgl2*, *Δcbp*	This study
TH6	*T. heterothallica*, *Δbgl1*, *Δbgl2*, *Δbgl3*, *Δbgl4*, *Δbgl5*, *Δcbp*	This study
TH9	*T. heterothallica*, *Δbgl1*, *Δbgl2*, *Δbgl3*, *Δbgl4*, *Δbgl5*, *Δbgl6*, *Δbgl7*, *Δbgl8*, *Δcbp*	This study
TH11	*T. heterothallica*, *Δbgl1*, *Δbgl2*, *Δbgl3*, *Δbgl4*, *Δbgl5*, *Δbgl6*, *Δbgl7*, *Δbgl8*, *Δcbp*, *Δcbt1*, *Δcbt2*	This study

**Fig. 2:**
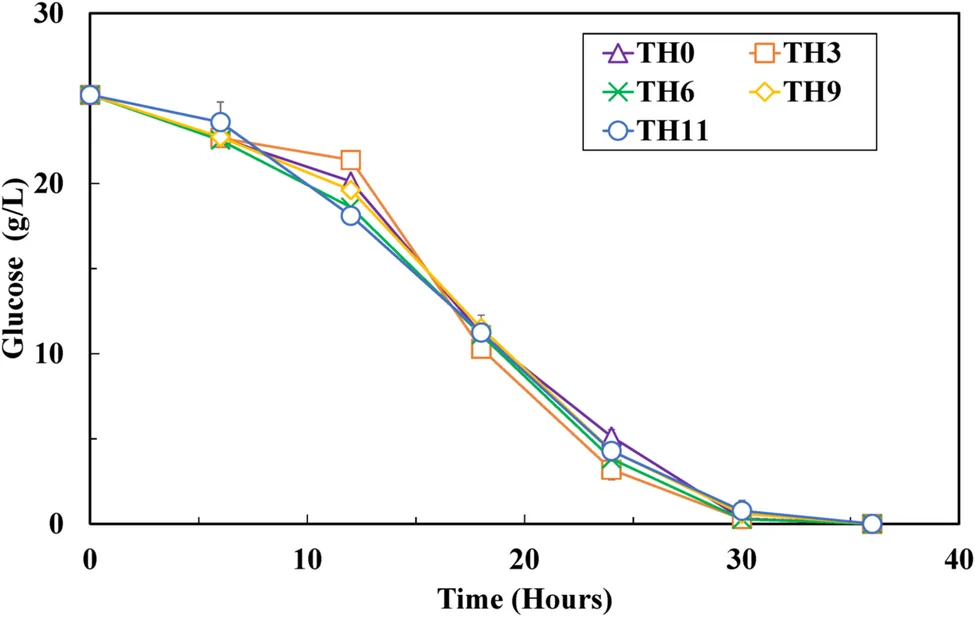
Glucose utilization by parental strain (TH0) and engineered strains (TH3, TH6, TH9, and TH11). Strains were cultivated in shake flasks containing 25 g/L glucose. Data represent mean ± SD of triplicate cultures

The parental and engineered strains were compared for their ability to utilize glucose to determine whether the multiple gene knockouts affected general carbon metabolism and growth. As shown in Fig. 2, all strains had similar glucose utilization patterns. They consumed about 25 g/L glucose in about 30 h. It appears that multiple deletions of *bgl*, *cbp*, and *cbt* genes did not affect glucose utilization of these strains.

### Effect of cellobiose phosphorylase knockout on cellobionate utilization

**Fig. 3:**
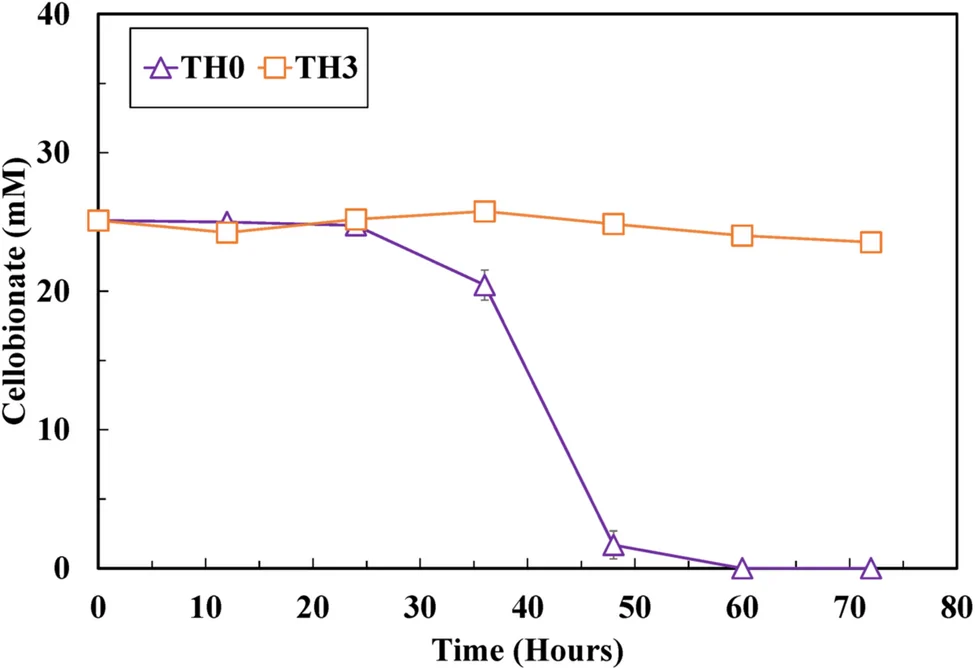
Effect of cellobiose phosphorylase knockout on cellobionate utilization. Cellobionate consumption by the parental strain TH0 and the triple-knockout strain TH3 (*Δbgl1*, *Δbgl2*, *Δcbp*) was evaluated using cellobionate as the substrate. Data represent mean ± SD of triplicate cultures

To evaluate the role of the putative cellobionate phosphorylase in cellobionate catabolism, cellobionate utilization was compared between the parental strain (TH0) and the triple deletion strain (TH3), in which two putative β-glucosidase genes (*bgl1* and *bgl2*) and one putative cellobionate phosphorylase gene were deleted. When cellobionate was provided as the substrate, TH0 rapidly consumed the supplemented cellobionate, with the concentration decreasing from approximately 25 mM to 0 mM within 60 h, as shown in Fig. 3. In contrast, strain TH3 retained nearly all of the supplied cellobionate throughout the experiment, maintaining concentrations of approximately 23–25 mM over the same period (Fig. 3). These results confirmed that knocking out the putative cellobionate phosphorylase gene successfully abolished cellobionate utilization.

In the *T. heterothallica* genome, the putative cellobionate phosphorylase gene shares 76.95% sequence identity and 88.79% sequence similarity with the characterized cellobionate phosphorylase from *N. crassa* (NCU09425). Cellobionate phosphorylase catalyzes phosphorolysis of cellobionate into glucose-1-phosphate and gluconic acid. (Nihira et al. 2013), representing the first intracellular step of cellobionate catabolism*.* Consistent with our findings, knocking out the corresponding cellobionate phosphorylase gene in *N. crassa* also eliminated cellobionate utilization (Hildebrand et al. 2015).

### Cellobionate production using cellobiose as substrate

**Fig. 4:**
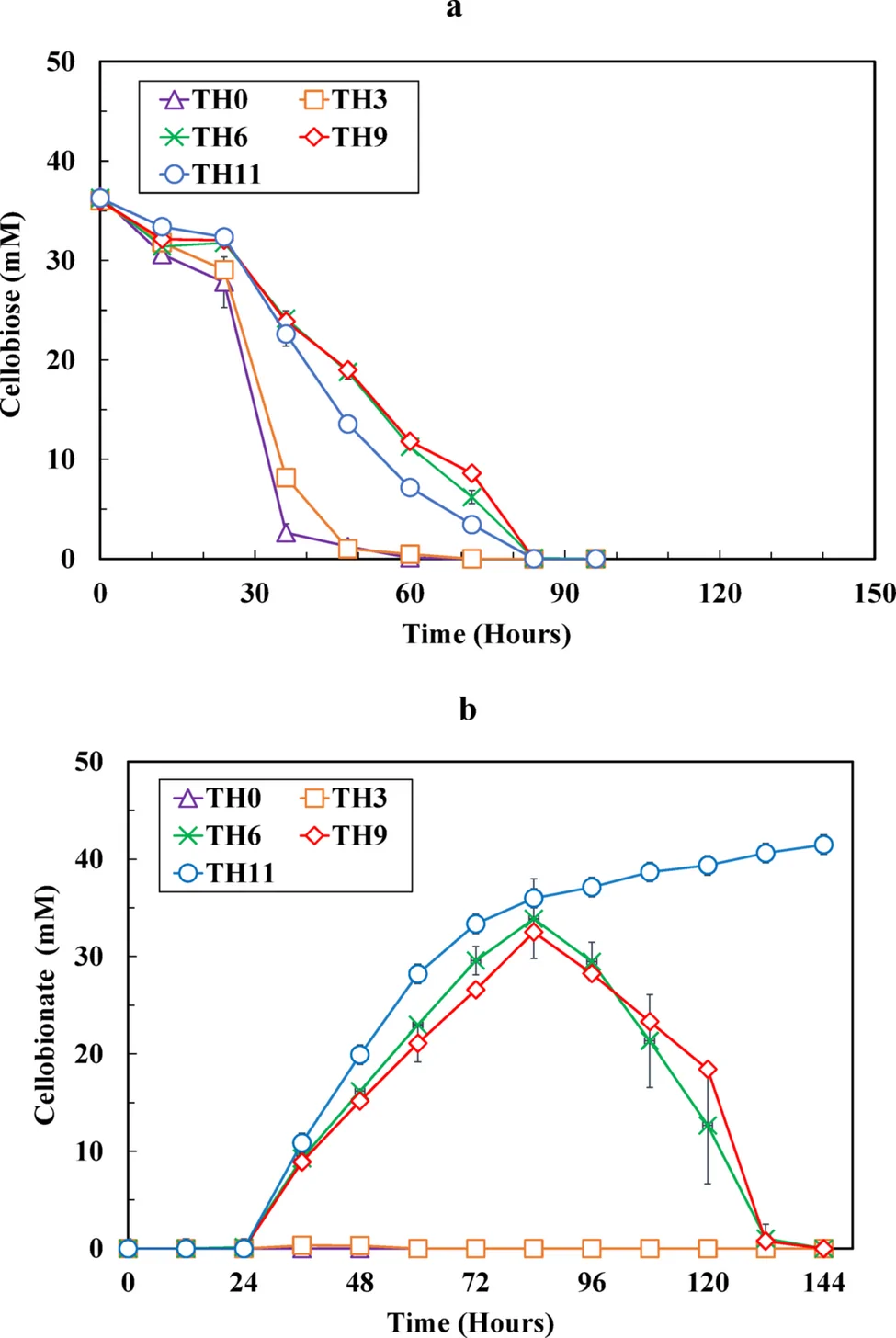
Cellobiose utilization and cellobionate production by parental and engineered strains using cellobiose as the substrate. **a** Residual cellobiose concentration and **b** cellobionate concentration during fermentation by strains TH0, TH3, TH6, TH9, and TH11. Data represent mean ± SD of triplicate cultures

To evaluate the effects of the targeted gene deletions on cellobiose metabolism and cellobionate production, the parental and engineered strains were cultivated using cellobiose as the substrate. As shown in Fig. 4a, substantial differences in cellobiose utilization and cellobionate production were observed among the strains. Strains TH0 and TH3 rapidly consumed 36 mM cellobiose within 60 h, but did not accumulate detectable levels of cellobionate. In contrast, strains TH6, TH9, and TH11 utilized cellobiose at slower rates than TH0 and TH3. The cellobiose consumption rates of TH9 and TH11 were nearly identical and slightly lower than that of TH6. Nevertheless, all strains completely consumed the cellobiose within 84 h. TH6 and TH9 produced cellobionate, reaching peak concentrations of approximately 33.9 mM and 32.5 mM at 84 h (Fig. 4b), respectively. These results suggest that sequential disruption of β-glucosidase genes reduced the intracellular conversion of cellobiose to glucose, thereby increasing the availability of cellobiose for oxidation by cellobiose dehydrogenase. However, after cellobiose was depleted, cellobionate concentrations in both strains gradually decreased to nearly undetectable levels by 144 h, indicating that the accumulated cellobionate was subsequently consumed or degraded. This observation suggests that cellobionate catabolism remained active in these strains despite the knockout of the cellobiose phosphorylase genes.

In contrast, strain TH11 continuously accumulated cellobionate throughout the fermentation, reaching 36 mM at 84 h (Fig. 4b), while cellobiose was completely depleted (Fig. 4a). Notably, the accumulated cellobionate remained stable even after cellobiose was exhausted.

Strain TH11 was derived from strain TH9, which already carried deletions of nine β-glucosidase genes, with the additional deletion of two putative cellobionate transporter genes, CBT1 (MYCTH_84164) and CBT2 (MYCTH_2302958). These results indicate that disruption of CBT1 and CBT2 was critical for preventing cellobionate consumption during the later stages of fermentation.

Sequence analysis showed that CBT1 (MYCTH_84164) of *T. heterothallica* shares 51.22% sequence identity and 68.29% sequence similarity with the *N. crassa* cellobionate transporter CBT1 (NCU05853), while CBT2 (MYCTH_2302958) shares 54.1% sequence identity and 72.0% sequence similarity with the same transporter. In *T. heterothallica*, CBT1 (MYCTH_84164) is annotated as a sugar transporter-like protein, whereas CBT2 (MYCTH_2302958) is classified as an uncharacterized protein. Nevertheless, the phenotypic results obtained in this study indirectly support their functional roles as cellobionate transporters.

When cellobiose was used as the substrate, the multiple β-glucosidase deletion strains of *T. heterothallica* (TH6, TH9, and TH11) were able to efficiently convert cellobiose to cellobionate (Fig. 4a), whereas the *N. crassa* heptuple β-glucosidase mutant could not effectively utilize cellobiose (Wu et al. 2013). The oxidation of cellobiose to cellobionate is catalyzed by cellobiose dehydrogenase, an enzyme typically co-regulated with cellulase expression and subject to carbon catabolite repression (Tian et al. 2009). In *N. crassa*, the presence of cellobiose represses cellulase production due to carbon catabolite repression, likely limiting the expression of cellobiose dehydrogenase and consequently restricting cellobionate formation. In contrast, the *T. heterothallica* mutants used in this study were derived from strain *T. heterothallica* C1, which carries a mutation in the global carbon catabolite repression regulator *cre−1*, thereby alleviating carbon catabolite repression (Visser et al. 2011). As a result, cellobiose dehydrogenase is likely expressed even in the presence of readily metabolizable carbon sources such as cellobiose, enabling efficient oxidation of cellobiose to cellobionate.

### Cellobionate production using pretreated wheat straw as substrate

**Fig. 5:**
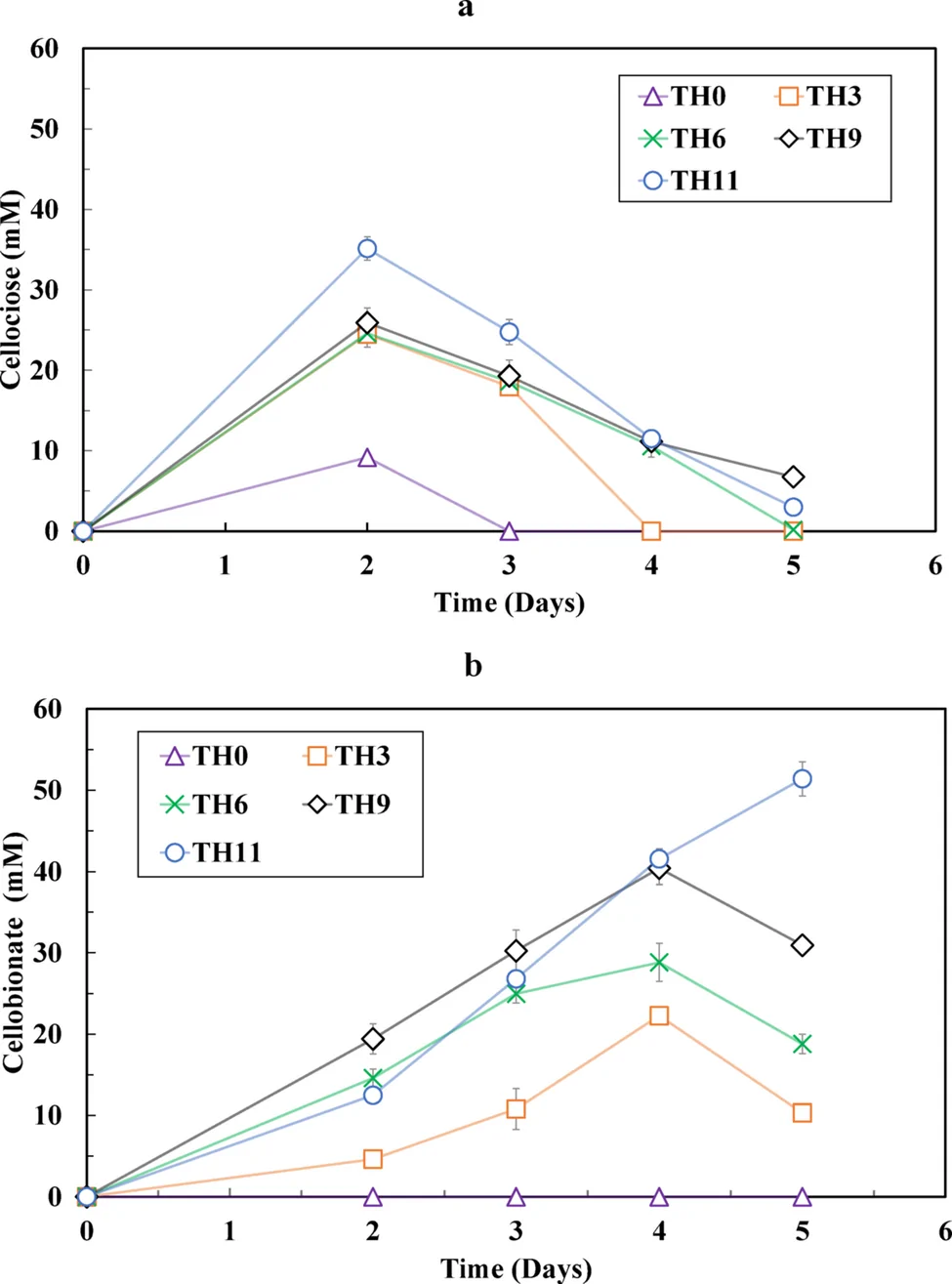
Cellobionate and cellobiose production from 2% NaOH-pretreated wheat straw by parental and engineered strains. **a** Cellobiose concentration and **b** cellobionate concentration during fermentation by strains TH0, TH3, TH6, TH9, and TH11. Data represent mean ± SD of triplicate cultures

The engineered strains were evaluated for cellobionate production using 2% pretreated wheat straw as the lignocellulosic substrate. As shown in Fig. 5b, the parental strain TH0 did not produce detectable levels of cellobionate from wheat straw, whereas all engineered strains were capable of producing cellobionate. Moreover, cellobionate production increased progressively with successive rounds of gene knockout. The maximum cellobionate titers achieved by strains TH3, TH6, TH9, and TH11 were 22.3 mM, 28.8 mM, 40.4 mM, and 51.4 mM, respectively (Fig. 5b).

Consistent with the results obtained using cellobiose as the substrate, the cellobionate concentrations in TH3, TH6, and TH9 cultures increased initially, reached peak levels, and then gradually declined during the later stages of fermentation, suggesting that these strains were able to further consume cellobionate (Fig. 5b). In contrast, the cellobionate concentration in the TH11 culture continued to increase until cellobiose was completely depleted, resulting in the highest cellobionate production among all strains tested (Fig. 5b).

During fermentation, *T. heterothallica* produced cellulases that hydrolyzed cellulose into cellobiose without the addition of exogenous enzymes. Cellobiose accumulated in the culture broth, reached a peak concentration, and was subsequently consumed over time (Fig. 5a). This accumulation pattern indicates that cellulose hydrolysis and cellobiose release occurred more rapidly than the subsequent oxidation of cellobiose to cellobionate, suggesting that the oxidation step was rate-limiting in the overall conversion process.

### Effect of pH control on cellobionate production by strain TH11

**Fig. 6:**
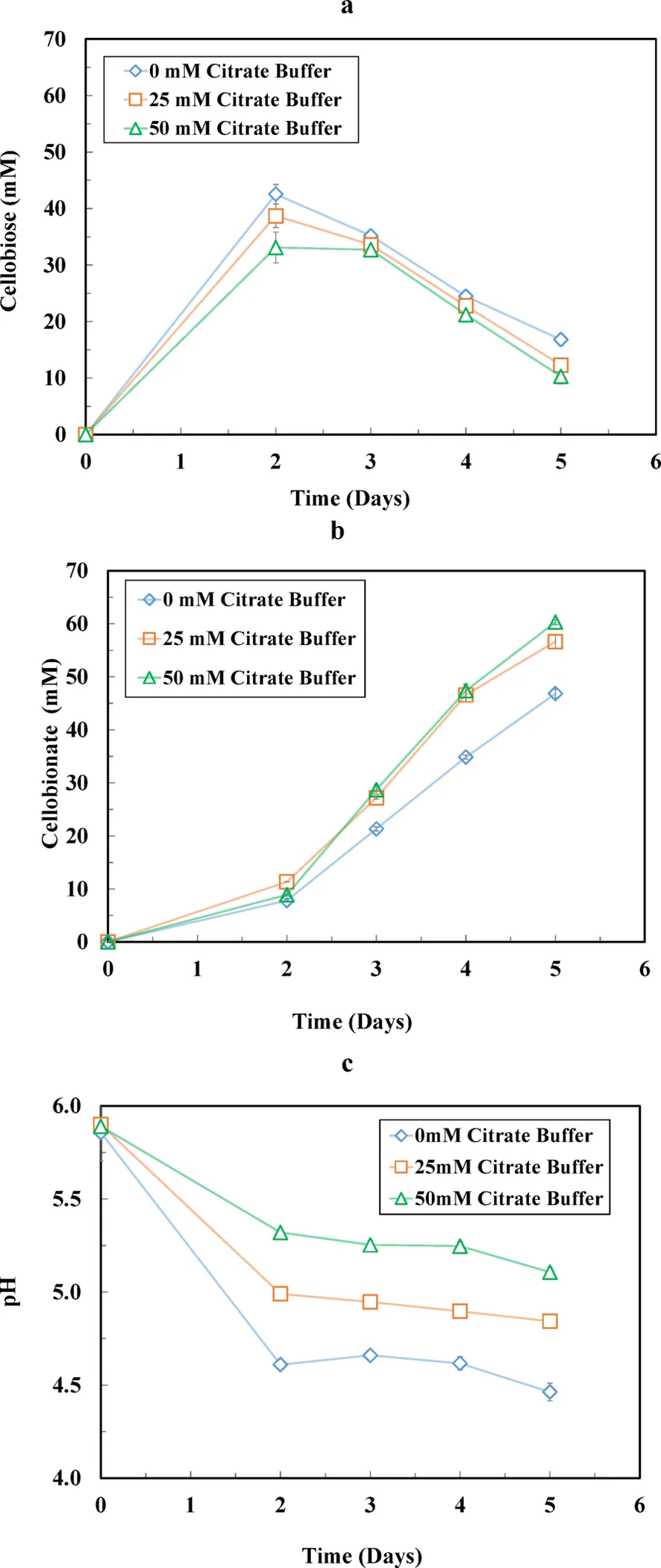
Effect of citrate buffer addition on cellobiose and cellobionate production by strain TH11 grown on 2% NaOH-pretreated wheat straw. Fermentations were carried out without buffer, with 25 mM citrate buffer (pH 6.0), or with 50 mM citrate buffer (pH 6.0). **a** Cellobiose concentration and **b** cellobionate concentration. **c** pH changes. Data represent mean ± SD of triplicate cultures

Since the production of CBA could decrease the pH of the medium and affect cellobionate production, the effect of pH control was evaluated by adding buffer solutions to the TH11 culture using pretreated wheat straw as the substrate (Fig. 6). Buffer addition improved cellobionate production. Without citrate buffer addition, strain TH11 produced about 46.8 mM cellobionate by day 5 (Fig. 6b). Addition of 25 mM citrate buffer (pH 6.0) to the initial fermentation broth slightly improved cellobionate production to 56.6 mM, whereas addition of 50 mM citrate buffer (pH 6.0) to the initial fermentation broth further increased the final cellobionate concentration to 60.3 mM (Fig. 6b). It appears that the citrate buffer enhances cellobionate production by helping maintain a more stable pH during fermentation. As shown in Fig. 6c, the pH of the fermentation broth without buffer addition decreased to approximately 4.48 from the initial pH of 6.0. In contrast, supplementation with 25 or 50 mM citrate buffer maintained the pH at approximately 4.8 and 5.1, respectively. The improved pH stability likely provided more favorable conditions for cellular metabolic activity and, consequently, enhanced cellobionate formation.

### Effect of temperature on CBA production by the strain TH11

**Fig. 7:**
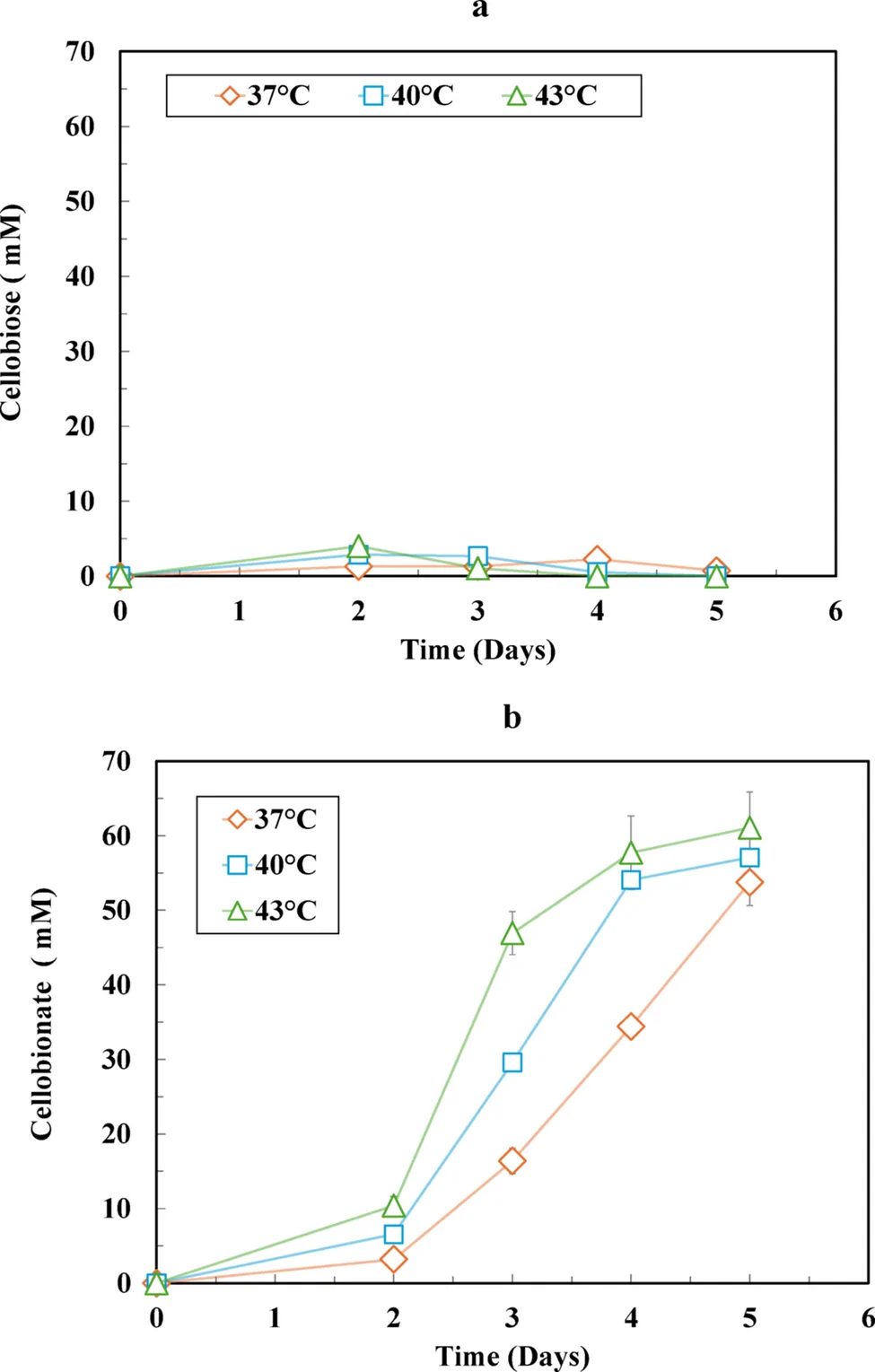
Effect of temperature on cellobiose and cellobionate production by strain TH11 grown on 2% NaOH-pretreated wheat straw. Fermentations were performed at 37, 40, and 43 °C. **a** Cellobiose concentration and **b** cellobionate concentration. Data represent mean ± SD of triplicate cultures

The effect of temperature on cellobiose production and its subsequent conversion into CBA by the strain TH11 was evaluated at 37, 40, and 43 °C using alkali-pretreated wheat straw as the substrate (Fig. 7). At 37 °C, cellobionate titer gradually increased and reached a maximal titer of 53.8 mM on day 5 (Fig. 7b). Increasing the temperature to 40 °C and 43 °C accelerated cellobionate production as compared to 37 °C. The highest performance was observed at 43 °C, where the cellobionate titer produced was visibly greater by day 3 compared to other conditions (Fig. 7b)**,** and the overall conversion was also slightly improved. The cellobiose was completely consumed by day 4, while some cellobiose was still left on day 4 when the fermentation was carried out at 37 and 40 °C (Fig. 7a). These results demonstrate that increasing temperature enhanced both the rate and titer of cellobionate production. Among the tested conditions, 43 °C was the optimal temperature, resulting in the fastest production kinetics and the highest final cellobionate titer. Fermentation at 46 °C did not further improve cellobionate production (Figure S1).

### Cellobionate production and enzyme activities under optimized conditions

**Fig. 8:**
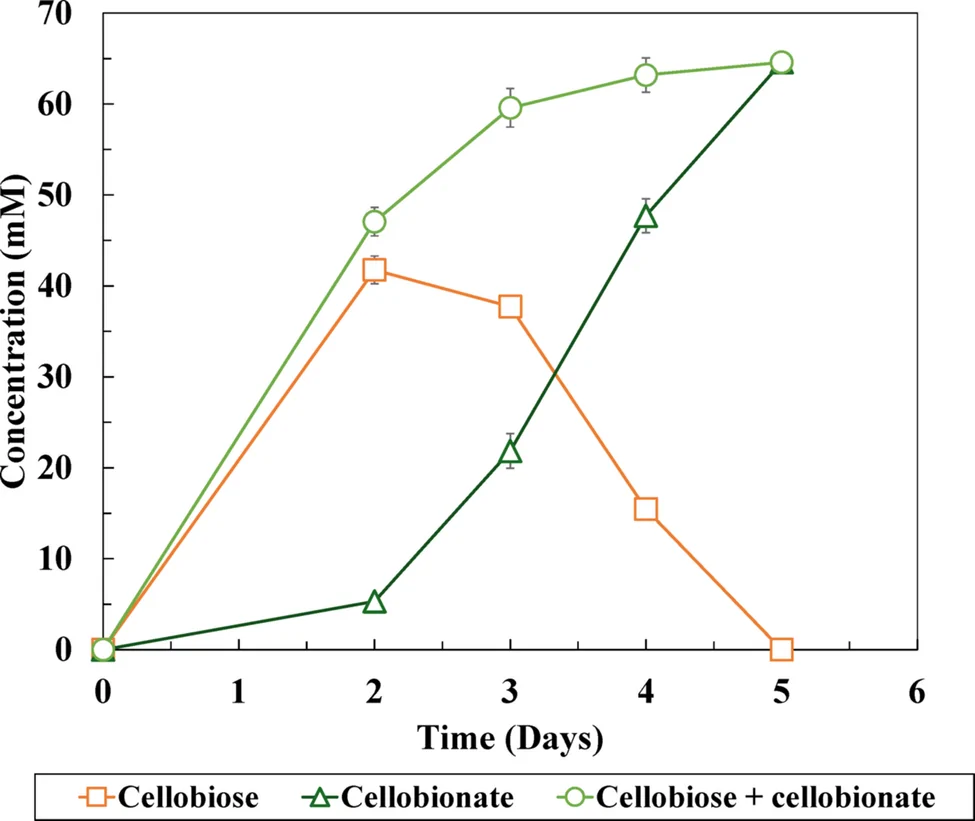
Production of cellobiose and cellobionate by strain TH11 under optimized conditions (43 °C, 50 mM citrate buffer, pH 6.0) using 2% (w/v) NaOH-pretreated wheat straw as substrate. Data represent mean ± SD of triplicate cultures

Under the optimized conditions of 43 °C in 50 mM citrate buffer (pH 6), fermentation of 2% pretreated wheat straw was carried out using strain TH11. As shown in Fig. 8, cellobiose rapidly accumulated to approximately 42 mM by day 2, while only a small amount of it was produced during the early stage of fermentation. After day 2, the cellobionate concentration increased continuously, whereas the cellobiose concentration gradually decreased, indicating that the accumulated cellobiose was subsequently converted into cellobionate. By day 5, cellobiose was completely consumed, and the cellobionate concentration reached approximately 64 mM. During this process, CBH, the primary cellulase enzyme responsible for cellobiose production, and CDH, which catalyzes the oxidation of cellobiose to cellobionate, were characterized. CBH activity increased progressively over the course of fermentation. Likewise, CDH activity exhibited a continuous upward trend over time, indicating sustained enzyme production and catalytic activity during cellobionate biosynthesis.

**Fig. 9:**
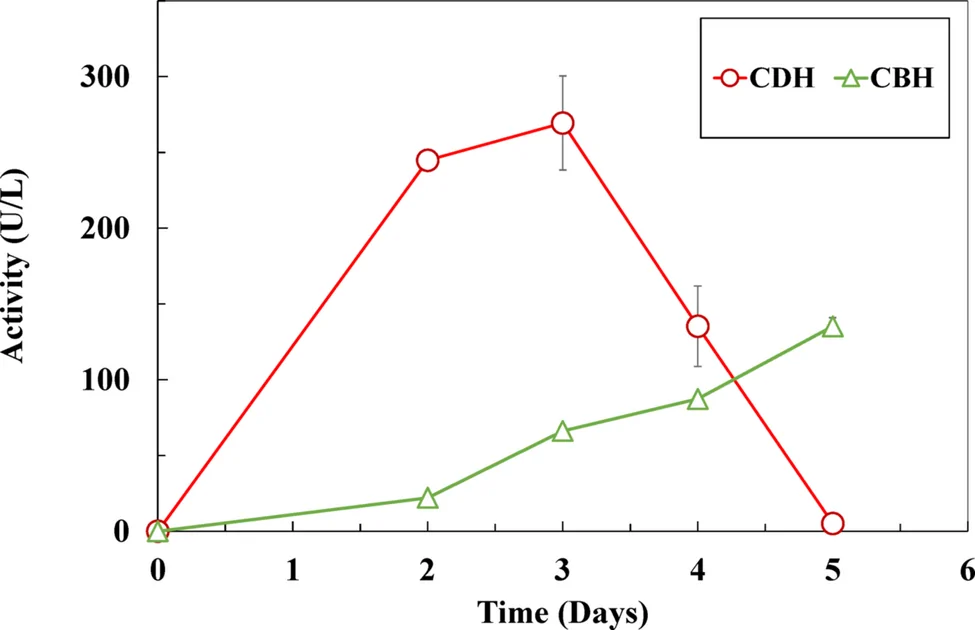
Extracellular cellobiohydrolase (CBH) and cellobiose dehydrogenase (CDH) activities produced by strain TH11 during fermentation of 2% NaOH-pretreated wheat straw under optimized conditions (43 °C, 50 mM citrate buffer, pH 6.0). Data represent mean ± SD of triplicate cultures

The combined concentration of cellobiose and cellobionate, which reflects the overall extent of cellulose hydrolysis, reached 59.6 mM by day 3 and gradually increased to 64.6 mM thereafter (Fig. 8). However, considering the concentration effect caused by evaporation during fermentation, cellulose hydrolysis appeared to have largely plateaued by day 3, while the later increase in cellobionate mainly resulted from continued conversion of accumulated cellobiose into cellobionate. During the process of fermentation, CDH and CBH activities were tracked. As shown in Fig. 9, CBH activities continued to increase throughout the fermentation period, reaching a maximum of 135 U/L. In contrast, CDH activity peaked at approximately 441 U/L on Day 3, and subsequently declined.

To accurately assess substrate conversion and product yield, a separate experiment was performed under the same conditions and harvested after 5 days without intermediate sampling. The supernatants were analyzed for cellobiose and cellobionate concentrations, whereas the residual solids were subjected to compositional analysis to determine the remaining cellulose and xylan contents. As shown in Table 2, cellulose and xylan conversions were 88.0% and 95.6%, respectively. The cellobionate yield from the consumed cellulose reached 96.2%, indicating highly efficient bioconversion of cellulose to cellobionate.

**Table 2:** Percentages of cellulose hydrolyzed and converted to cellobionate by strain TH11 grown on 20 g/L cellulose equivalent of NaOH-pretreated wheat straw

Fermentation condition	Amount (g) of cellulose		Cellulose conversion (%)	Xylan conversion (%)	Yield (%) from consumed cellulose
	Starting	Residual			
TH11, 43 °C, 50 mM citrate buffer, pH 6.0	1.0	0.12 ± 0.01	87.9 ± 0.8	95.6 ± 2.8	96.2 ± 1.5

The *N. crassa*, strain HL10 required approximately 8 days to produce 57 mM cellobionate, whereas the *T. heterothallica* TH11, engineered in this study, produced 64 mM cellobionate within only 5 days. The cellulose conversion and cellobionate yield from consumed cellulose were comparable between the two strains. In addition, the CDH and CBH activities detected during fermentation were higher than those observed for *N. crassa* HL10 within the same fermentation period. The higher productivity achieved by strain TH11 highlights the advantage of using a thermophilic cellulolytic fungal host for direct lignocellulose bioconversion. Compared with mesophilic systems, thermophilic fermentation at 43 °C likely improved hydrolysis efficiency and the enzymatic reaction kinetics. Elevated temperature may also reduce contamination risks and improve process robustness during large-scale operation. Importantly, *T. heterothallica* naturally secretes high levels of extracellular cellulases, allowing efficient biomass deconstruction without supplementation of external enzyme cocktails. This feature represents a major economic advantage for lignocellulosic biorefineries, where enzyme costs remain one of the major barriers to commercialization.

Despite the high conversion efficiency achieved in this study, the transient accumulation of cellobiose indicates that the process is oxidation-limited. Process optimization in oxygen supply using a bioreactor, together with additional engineering of oxidative metabolism, may further improve productivity.

## Conclusions

In this study, *T. heterothallica* was successfully engineered for direct cellobionate production from alkali-pretreated wheat straw without any enzyme addition. Sequential knockout of β-glucosidase, cellobionate phosphorylase, and putative cellobionate transporter genes effectively redirected carbon flux toward cellobionate production and prevented product re-consumption. The engineered *T. heterothallica* strain, TH11, produced approximately 64–66 mM cellobionate with high cellulose conversion. Compared with the previously engineered *N. crassa* strain, the *T. heterothallica* TH11 exhibited faster production kinetics and higher enzymatic activities. To our knowledge, this is the first demonstration of an engineered thermophilic filamentous fungus capable of directly converting pretreated lignocellulosic biomass to cellobionate without the addition of exogenous cellulases. This consolidated bioprocessing capability integrates cellulose hydrolysis and product formation in a single microbial system, offering an important advantage over conventional enzyme-dependent biomass conversion processes.

## Supplementary Information

Below is the link to the electronic supplementary material.

Supplementary Material 1

## Data Availability

All data generated or analyzed during this study are included in the published article or in the supplementary information.

## Abbreviations

CBA: Cellobionic acid/Cellobionate
IMM: Isotonic/Isoosmotic minimal medium
RMYE: Rich medium with yeast extracts
RNP: Ribonucleoprotein
PEG: Polyethylene glycol
STC: Sorbitol–Tris–Calcium chloride
CBH: Cellobiohydrolase
CDH: Cellobiose dehydrogenase
DCPIP: 2,6-Dichlorophenolindophenol
CBAP: Cellobiose phosphorylase
pNPL: *p*-Nitrophenyl-β-D-lactoside
