# Supplementary material for: Engineering Thermothelomyces heterothallica for cellobionate production
Source: Bioresour Bioprocess. 2026 Sep 3;13(1):129. doi: 10.1186/s40643-026-01126-0 (PMC13542005; doi:10.1186/s40643-026-01126-0)
Supplement: Supplementary file 1 [file 40643_2026_1126_MOESM1_ESM.docx]

**Metabolic Engineering of *Thermothelomyces heterothallica* C1 for Cellobionate Production**

Bakht Zada^1†^, Hamidreza Shapouri^1†^, Jiajie Wang^1^, Sumit Sharma^1^, Takao Kasuga^2,3^, Noelia Valbuena^4^, Ronen Tchelet^4^, Mark Emalfarb^4^, Zhiliang Fan^*1^

**Table S1. Primers used in this study.**

| **Names** | **Descriptions** | **References** |
| --- | --- | --- |
| **Primers** |  |  |
| Hyg-F | AATTCCCTTGTATCTCTACACACAGG | This study |
| Hyg-R | CCTCTAAACAAGTGTACCTGTGC | This study |
| Bar-F | GACCTATACTAGACTCGACAG | This study |
| Bar-R | GGAGCTTGAGATCCACTTAAC | This study |
| Bar-CDS-F | AGGAAGTAACCATGAGCCCAG | This study |
| Bar-CDS-R | GCCAAATGTTTGAACGATCTGC | This study |
| Neo-F | AATTCCCTTGTATCTCTACACACAGG | This study |
| Neo-R | CCTCTAAACAAGTGTACCTGTGC | This study |
| Neo_CDS-F | ATGATTGAACAAGATGGATTGCAC | This study |
| Neo_CDS-R | TCAGAAGAACTCGTCAAGAAGGC | This study |
| Zeo-F | TGACTTGAGCCTAAAATCCGC | This study |
| Zeo-R | AGCTTGCAAATTAAAGCCTTCG | This study |
| Bgl1-F | CAGACAATCGTCCATCGTCC | This study |
| Bgl1-R | CATACTCCAACTCCTGCCTGC | This study |
| Bgl2-F | CACACGCCGAAATTTACGAC | This study |
| Bgl2-R | CTGCAACGACGCTGTCCG | This study |
| CBP-F | TGTTGGCCCCTCCACAGG | This study |
| CBP-R | CCTCACTGCAACAACACCG | This study |
| Bgl3-F | CTCCTTATCCCAACGCCG | This study |
| Bgl3-R | CCGTTCCACAACTCGGGAC | This study |
| Bgl4-F | AGCAGGTCGTGAGAAGGG | This study |
| Bgl4-R | CCACGCTGCCGCTATTAAATG | This study |
| Bgl5-F | GTGATCGAGGTTTCACGACCC | This study |
| Bgl5-R | GCTCCTGCCTAGCCCTAAC | This study |
| Bgl6-F | AACAGCAAGGCGCTCAAC | This study |
| Bgl6-R | CACCTACCGCAAGAGCCT | This study |
| Bgl7-F | TTCCGAGACTCGCCAAGC | This study |
| Bgl7-R | GGGACCGACCCTTGAGTG | This study |
| Bgl8-F | CCTCGTATGGCCTTGGAC | This study |
| Bgl8-R | ACCGCAGAGGTGTCTTACC | This study |
| CBT1-F | TTCGACAGCGTCCAGACG | This study |
| CBT1-R | TACCTGAGGCGCCTTCAG | This study |
| CBT2-F | TCCGACTCGACTCTGTCC | This study |
| CBT2-R | CCCAACCCCGGGTATATTG | This study |

**Table S2. CRISPR Cas9-crRNAs used in this study.**

| **Names** | **Descriptions** | **References** |
| --- | --- | --- |
| **CRISPR crRNA** |  |  |
| Bgl1-crRNA-1 | AGTCGTGAAAGTACTTGACG | This study |
| Bgl1-crRNA-2 | GAGGACTTCAAGCCCACGCA | This study |
| Bgl2-crRNA-1 | ACCTCCTCAAGAACGAGCTT | This study |
| Bgl2-crRNA-2 | TGGTCTTGGTGACCTTGAAG | This study |
| Bgl3-crRNA-1 | ACCTTGCGGATGCTGTCCAG | This study |
| Bgl3-crRNA-2 | GGGCTACGACCGCAAGAGCA | This study |
| Bgl4-crRNA-1 | AGTCTGACATGACCATGCCC | This study |
| Bgl4-crRNA-2 | GAGTTCCCGCTCTTCTTCAC | This study |
| Bgl5-crRNA-1 | GTTGTAGGAGCACATGACGG | This study |
| Bgl5-crRNA-2 | AAGGTCATGTACCTGCCAAA | This study |
| Bgl6-crRNA-1 | TTCCGGTCAACACTGTTCGA | This study |
| Bgl6-crRNA-2 | CATCGACCACCCCAACGTGA | This study |
| Bgl7-crRNA-1 | GGACAAGATCAACATCGTGA | This study |
| Bgl7-crRNA-2 | TCAACGGAGGGACGATTCTC | This study |
| Bgl8-crRNA-1 | CGTCAACCAGCTCTTTGCCC | This study |
| Bgl8-crRNA-2 | CATCGTCAAGTACATCCTCC | This study |
| CBP-crRNA-1 | AAAGTTGTCGAGCTCCTTGT | This study |
| CBP-crRNA-2 | AAGATCACCGAGTTCAAGGC | This study |
| CBT1-crRNA-1 | CTTTATCGGAGCCATCGTGG | This study |
| CBT1-crRNA-2 | TTGAAGCACAGGTTGTACGC | This study |
| CBT2-crRNA-1 | TGCAGGAGGACAAGATCCCA | This study |
| CBT2-crRNA-2 | AGATGGCCATGTACTTCCAG | This study |

**Table S3. Genes targeted for metabolic engineering in this study.**

| **Gene** | **Gene ID** | **Locus Tag** | **Encoded Protein** | **Subcellular Localization** |
| --- | --- | --- | --- | --- |
| *cbp/cbap* | 11506721 | MYCTH_2308030 | Cellobiose/cellobionate phosphorylase | Intracellular |
| *bgl1* | 11512780 | MYCTH_115968 | β-glucosidase | Intracellular |
| *bgl2* | 11509376 | MYCTH_80304 | β-glucosidase | Extracellular |
| *bgl3* | 11505760 | MYCTH_62925 | β-glucosidase | Intracellular |
| *bgl4* | 11512162 | MYCTH_2059579 | β-glucosidase | Extracellular |
| *bgl5* | 11506369 | MYCTH_38200 | β-glucosidase | Intracellular |
| *bgl6* | 11512244 | MYCTH_2302509 | β-glucosidase | Intracellular |
| *bgl7* | 11509700 | MYCTH_66804 | β-glucosidase | Extracellular |
| *bgl8* | 11511513 | MYCTH_58882 | β-glucosidase | Extracellular |
| *cbt1* | 11506621 | MYCTH_84164 | CBA Transporter | Plasma membrane |
| *cbt2* | 11512434 | MYCTH_2302958 | CBA Transporter | Plasma membrane |

**Table S4. Nucleotide sequence of antibiotic markers used for strains engineering in this study.**

| **Antibiotic Cassette** | **Nucleotide Sequence** |
| --- | --- |
| Hygromycin | AATTCCCTTGTATCTCTACACACAGGCTCAAATCAATAAGAAGAACGGTTCGTCTTTTTCGTTTATATCTTGCATCGTCCCAAAGCTATTGGCGGGATATTCTGTTTGCAGTTGGCTGACTTGAAGTAATCTCTGCAGATCTTTCGACACTGAAATACGTCGAGCCTGCTCCGCTTGGAAGCGGCGAGGAGCCTCGTCCTGTCACAACTACCAACATGGAGTACGATAAGGGCCAGTTCCGCCAGCTCATTAAGAGCCAGTTCATGGGCGTTGGCATGATGGCCGTCATGCATCTGTACTTCAAGTACACCAACCCTCTTCTGATCCAGTCGATCATCCCGCTGAAGGGCGCTTTCGAATCGAATCTGGTTAAGATCCACGTCTTCGGGAAGCCAGCGACTGGTGACCTCCAGCGTCCCTTTAAGGCTGCCAACAGCTTTCTCAGCCAGGGCCAGCCCAAGACCGACAAGGCCTCCCTCCAGAACGCCGAGAAGAACTGGAGGGGTGGTGTCAAGGAGGAGTAAGCTCCTTATTGAAGTCGGAGGACGGAGCGGTGTCAAGAGGATATTCTTCGCTCTGTATTATAGATAAGATGATGAGGAATTGGAGGTAGCATAGCTTCATTTGGATTTGCTTTCCAGGCTGAGACTCTAGCTTGGAGCATAGAGGGTCCCTTTGGCTTTCAATATTCTCAAGTATCTCGAGTTTGAACTTATTCCCGTGAACCTTTTATTCACCAATGAGCATTGGAATGAACATGAATCTGAGGACTGCAATCGCCATGAGGTTTTCGAAATACATCCGGATGTCGAAGGCTTGGGGCACCTGCGTTGGTTGAATTTAGAACGTGGCACTATTGATCATCCGATAGCTCTGCAAAGGGCGTTGCACAATGCAAGTCAAACGTTGCTAGCAGTTCCAGGTGGAATGTTATGATGAGCATTGTATTAAATCAGGAGATATAGCATGATCTCTAGTTAGCTCACCACAAAAGTCAGACGGCGTAACCAAAAGTCACACAACACAAGCTGTAAGGATTTCGGCACGGCTACGGAAGACGGAGAAGCCCACCTTCAGTGGACTCGAGTACCATTTAATTCTATTTGTGTTTGATCGAGACCTAATACAGCCCCTACAACGACCATCAAAGTCGTATAGCTACCAGTGAGGAAGTGGACTCAAATCGACTTCAGCAACATCTCCTGGATAAACTTTAAGCCTAAACTATACAGAATAAGATGGTGGAGAGCTTATACCGAGCTCCCAAATCTGTCCAGATCATGGTTGACCGGTGCCTGGATCTTCCTATAGAATCATCCTTATTCGTTGACCTAGCTGATTCTGGAGTGACCCAGAGGGTCATGACTTGAGCCTAAAATCCGCCGCCTCCACCATTTGTAGAAAAATGTGACGAACTCGTGAGCTCTGTACAGTGACCGGTGACTCTTTCTGGCATGCGGAGAGACGGACGGACGCAGAGAGAAGGGCTGAGTAATAAGCGCCACTGCGCCAGACAGCTCTGGCGGCTCTGAGGTGCAGTGGATGATTATTAATCCGGGACCGGCCGCCCCTCCGCCCCGAAGTGGAAAGGCTGGTGTGCCCCTCGTTGACCAAGAATCTATTGCATCATCGGAGAATATGGAGCTTCATCGAATCACCGGCAGTAAGCGAAGGAGAATGTGAAGCCAGGGGTGTATAGCCGTCGGCGAAATAGCATGCCATTAACCTAGGTACAGAAGTCCAATTGCTTCCGATCTGGTAAAAGATTCACGAGATAGTACCTTCTCCGAAGTAGGTAGAGCGAGTACCCGGCGCGTAAGCTCCCTAATTGGCCCATCCGGCATCTGTAGGGCGTCCAAATATCGTGCCTCTCCTGCTTTGCCCGGTGTATGAAACCGGAAAGGCCGCTCAGGAGCTGGCCAGCGGCGCAGACCGGGAACACAAGCTGGCAGTCGACCCATCCGGTGCTCTGCACTCGACCTGCTGAGGTCCCTCAGTCCCTGGTAGGCAGCTTTGCCCCGTCTGTCCGCCCGGTGTGTCGGCGGGGTTGACAAGGTCGTTGCGTCAGTCCAACATTTGTTGCCATATTTTCCTGCTCTCCCCACCAGCTGCTCTTTTCTTTTCTCTTTCTTTTCCCATCTTCAGTATATTCATCTTCCCATCCAAGAACCTTTATTTCCCCTAAGTAAGTACTTTGCTACATCCATACTCCATCCTTCCCATCCCTTATTCCTTTGAACCTTTCAGTTCGAGCTTTCCCACTTCATCGCAGCTTGACTAACAGCTACCCCGCTTGAGCAGACATCACCATGAAAAAGCCTGAACTCACCGCGACGTCTGTCGAGAAGTTTCTGATCGAAAAGTTCGACAGCGTCTCCGACCTGATGCAGCTCTCGGAGGGCGAAGAATCTCGTGCTTTCAGCTTCGATGTAGGAGGGCGTGGATATGTCCTGCGGGTAAATAGCTGCGCCGATGGTTTCTACAAAGATCGTTATGTTTATCGGCACTTTGCATCGGCCGCGCTCCCGATTCCGGAAGTGCTTGACATTGGGGAATTCAGCGAGAGCCTGACCTATTGCATCTCCCGCCGTGCACAGGGTGTCACGTTGCAAGACCTGCCTGAAACCGAACTGCCCGCTGTTCTGCAGCCGGTCGCGGAGGCCATGGATGCGATCGCTGCGGCCGATCTTAGCCAGACGAGCGGGTTCGGCCCATTCGGACCGCAAGGAATCGGTCAATACACTACATGGCGTGATTTCATATGCGCGATTGCTGATCCCCATGTGTATCACTGGCAAACTGTGATGGACGACACCGTCAGTGCGTCCGTCGCGCAGGCTCTCGATGAGCTGATGCTTTGGGCCGAGGACTGCCCCGAAGTCCGGCACCTCGTGCACGCGGATTTCGGCTCCAACAATGTCCTGACGGACAATGGCCGCATAACAGCGGTCATTGACTGGAGCGAGGCGATGTTCGGGGATTCCCAATACGAGGTCGCCAACATCTTCTTCTGGAGGCCGTGGTTGGCTTGTATGGAGCAGCAGACGCGCTACTTCGAGCGGAGGCATCCGGAGCTTGCAGGATCGCCGCGGCTCCGGGCGTATATGCTCCGCATTGGTCTTGACCAACTCTATCAGAGCTTGGTTGACGGCAATTTCGATGATGCAGCTTGGGCGCAGGGTCGATGCGACGCAATCGTCCGATCCGGAGCCGGGACTGTCGGGCGTACACAAATCGCCCGCAGAAGCGCGGCCGTCTGGACCGATGGCTGTGTAGAAGTACTCGCCGATAGTGGAAACCGACGCCCCAGCACTCGTCCGAGGGCAAAGGAATAGAGTAGATGCCGACCGGGATCCACTTAACGTTACTGAAATCATCAAACAGCTTGACGAATCTGGATATAAGATCGTTGGTGTCGATGTCAGCTCCGGAGTTGAGACAAATGGTGTTCAGGATCTCGATAAGATACGTTCATTTGTCCAAGCAGCAAAGAGTGCCTTCTAGTGATTTAATAGCTCCATGTCAACAAGAATAAAACGCGTTTCGGGTTTACCTCTTCCAGATACAGCTCATCTGCAATGCATTAATGCATTGGACCTCGCAACCCTAGTACGCCCTTCAGGCTCCGGCGAAGCAGAAGAATAGCTTAGCAGAGTCTATTTTCATTTTCGGGAGACGAGATCAAGCAGATCAACGGTCGTCAAGAGACCTACGAGACTGAGGAATCCGCTCTTGGCTCCACGCGACTATATATTTGTCTCTAATTGTACTTTGACATGCTCCTCTTCTTTACTCTGATAGCTTGACTATGAAAATTCCGTCACCAGCCCCTGGGTTCGCAAAGATAATTGCACTGTTTCTTCCTTGAACTCTCAAGCCTACAGGACACACATTCATCGTAGGTATAAACCTCGAAAATCATTCCTACTAAGATGGGTATACAATAGTAACCATGGTTGCCTAGTGAATGCTCCGTAACACCCAATACGCCGGCCGAAACTTTTTTACAACTCTCCTATGAGTCGTTTACCCAGAATGCACAGGTACACTTGTTTAGAGG |
| Phosphinothricin | GACCTATACTAGACTCGACAGAAGATGATATTGAAGGAGCACTTTTTGGGCTTGGCTGGAGCTAGTGGAGGTCAACAATGAATGCCTATTTTGGTTTAGTCGTCCAGGCGGTGAGCACAAAATTTGTGTCGTTTGACAAGATGGTTCATTTAGGCAACTGGTCAGATCAGCCCCACTTGTAGCAGTAGCGGCGGCGCTCGAAGTGTGACTCTTATTAGCAGACAGGAACGAGGACATTATTATCATCTGCTGCTTGGTGCACGATAACTTGGTGCGTTTGTCAAGCAAGGTAAGTGAACGACCCGGTCATACCTTCTTAAGTTCGCCCTTCCTCCCTTTATTTCAGATTCAATCTGACTTACCTATTCTACCCAAGCAAAGCTTCGATTAGGAAGTAACCATGAGCCCAGAACGACGCCCGGCCGACATCCGCCGTGCCACCGAGGCGGACATGCCGGCGGTCTGCACCATCGTCAACCACTACATCGAGACAAGCACGGTCAACTTCCGTACCGAGCCGCAGGAACCGCAGGAGTGGACGGACGACCTCGTCCGTCTGCGGGAGCGCTATCCCTGGCTCGTCGCCGAGGTGGACGGCGAGGTCGCCGGCATCGCCTACGCGGGCCCCTGGAAGGCACGCAACGCCTACGACTGGACGGCCGAGTCGACCGTGTACGTCTCCCCCCGCCACCAGCGGACGGGACTGGGCTCCACGCTCTACACCCACCTGCTGAAGTCCCTGGAGGCACAGGGCTTCAAGAGCGTGGTCGCTGTCATCGGGCTGCCCAACGACCCGAGCGTGCGCATGCACGAGGCGCTCGGATATGCCCCCCGCGGCATGCTGCGGGCGGCCGGCTTCAAGCACGGGAACTGGCATGACGTGGGTTTCTGGCAGCTGGACTTCAGCCTGCCGGTACCGCCCCGTCCGGTCCTGCCCGTCACCGAGATCTGATCCGTCGACCTGCAGATCGTTCAAACATTTGGCAATAAAGTTTCTTAAGATTGAATCCTGTTGCCGGTCTTGCGATGATTATCATATAATTTCTGTTGAATTACGTTAAGCATGTAATAATTAACATGTAATGCATAGTACCGAGAAAAAGAAGGATTACCTCTAAACAAGTGTACCTGTGCATTCTGGGTAAACGACTCATAGGAGAGTTGTAAAAAAGTTTCGGCCGGCGTATTGGGTGTTACGGAGCATTCACTAGGCAACCATGCATGGTTACTATTGTATACCATCTTAGTAGGAATGATTTCGAGGTTTATACCTACGATGAATGTGTGTCCTGTAGGCTTGAGAGTTCAAGGAAGAAACATGCAATTATCTTTGCGAACCCAGGGGCTGGTGACGGAATTTTCATAGTCAAGCTATCAGAGTAAAGAAGAGGAGCATGTCAAAGTACAATTAGAGACAAATATATAGTCGCGTGGAGCCAAGAGCGGATTCCTCAGTCTCGTAGGTCTCTTGACGACCGTTGATCTGCTTGATCTCGTCTCCCGAAAATGAAAATAGCTCTGCTAAGCTATTCTTCTCTTCGCCGGAGCCTGAAGGCGTTACTAGGTTGCAGTCAATGCATTAATGCATTGCAGATGAGCTGTATCTGGAAGAGGTAAACCCGAAAACGCGTTTTATTCTTGTTGACATGGAGCTATTAAATCACTAGAAGGCACTCTTTGCTGCTTGGACAAATGAACGTATCTTATCGAGATCCTGAACACCATTTGTCTCAACTCCGGAGCTGACATCGACACCAACGATCTTATATCCAGATTCGTCAAGCTGTTTGATGATTTCAGTAACGTTAAGTGGATCTCAAGCTCC |
| Neomycin | AATTCCCTTGTATCTCTACACACAGGCTCAAATCAATAAGAAGAACGGTTCGTCTTTTTCGTTTATATCTTGCATCGTCCCAAAGCTATTGGCGGGATATTCTGTTTGCAGTTGGCTGACTTGAAGTAATCTCTGCAGATCTTTCGACACTGAAATACGTCGAGCCTGCTCCGCTTGGAAGCGGCGAGGAGCCTCGTCCTGTCACAACTACCAACATGGAGTACGATAAGGGCCAGTTCCGCCAGCTCATTAAGAGCCAGTTCATGGGCGTTGGCATGATGGCCGTCATGCATCTGTACTTCAAGTACACCAACCCTCTTCTGATCCAGTCGATCATCCCGCTGAAGGGCGCTTTCGAATCGAATCTGGTTAAGATCCACGTCTTCGGGAAGCCAGCGACTGGTGACCTCCAGCGTCCCTTTAAGGCTGCCAACAGCTTTCTCAGCCAGGGCCAGCCCAAGACCGACAAGGCCTCCCTCCAGAACGCCGAGAAGAACTGGAGGGGTGGTGTCAAGGAGGAGTAAGCTCCTTATTGAAGTCGGAGGACGGAGCGGTGTCAAGAGGATATTCTTCGCTCTGTATTATAGATAAGATGATGAGGAATTGGAGGTAGCATAGCTTCATTTGGATTTGCTTTCCAGGCTGAGACTCTAGCTTGGAGCATAGAGGGTCCCTTTGGCTTTCAATATTCTCAAGTATCTCGAGTTTGAACTTATTCCCGTGAACCTTTTATTCACCAATGAGCATTGGAATGAACATGAATCTGAGGACTGCAATCGCCATGAGGTTTTCGAAATACATCCGGATGTCGAAGGCTTGGGGCACCTGCGTTGGTTGAATTTAGAACGTGGCACTATTGATCATCCGATAGCTCTGCAAAGGGCGTTGCACAATGCAAGTCAAACGTTGCTAGCAGTTCCAGGTGGAATGTTATGATGAGCATTGTATTAAATCAGGAGATATAGCATGATCTCTAGTTAGCTCACCACAAAAGTCAGACGGCGTAACCAAAAGTCACACAACACAAGCTGTAAGGATTTCGGCACGGCTACGGAAGACGGAGAAGCCCACCTTCAGTGGACTCGAGTACCATTTAATTCTATTTGTGTTTGATCGAGACCTAATACAGCCCCTACAACGACCATCAAAGTCGTATAGCTACCAGTGAGGAAGTGGACTCAAATCGACTTCAGCAACATCTCCTGGATAAACTTTAAGCCTAAACTATACAGAATAAGATGGTGGAGAGCTTATACCGAGCTCCCAAATCTGTCCAGATCATGGTTGACCGGTGCCTGGATCTTCCTATAGAATCATCCTTATTCGTTGACCTAGCTGATTCTGGAGTGACCCAGAGGGTCATGACTTGAGCCTAAAATCCGCCGCCTCCACCATTTGTAGAAAAATGTGACGAACTCGTGAGCTCTGTACAGTGACCGGTGACTCTTTCTGGCATGCGGAGAGACGGACGGACGCAGAGAGAAGGGCTGAGTAATAAGCGCCACTGCGCCAGACAGCTCTGGCGGCTCTGAGGTGCAGTGGATGATTATTAATCCGGGACCGGCCGCCCCTCCGCCCCGAAGTGGAAAGGCTGGTGTGCCCCTCGTTGACCAAGAATCTATTGCATCATCGGAGAATATGGAGCTTCATCGAATCACCGGCAGTAAGCGAAGGAGAATGTGAAGCCAGGGGTGTATAGCCGTCGGCGAAATAGCATGCCATTAACCTAGGTACAGAAGTCCAATTGCTTCCGATCTGGTAAAAGATTCACGAGATAGTACCTTCTCCGAAGTAGGTAGAGCGAGTACCCGGCGCGTAAGCTCCCTAATTGGCCCATCCGGCATCTGTAGGGCGTCCAAATATCGTGCCTCTCCTGCTTTGCCCGGTGTATGAAACCGGAAAGGCCGCTCAGGAGCTGGCCAGCGGCGCAGACCGGGAACACAAGCTGGCAGTCGACCCATCCGGTGCTCTGCACTCGACCTGCTGAGGTCCCTCAGTCCCTGGTAGGCAGCTTTGCCCCGTCTGTCCGCCCGGTGTGTCGGCGGGGTTGACAAGGTCGTTGCGTCAGTCCAACATTTGTTGCCATATTTTCCTGCTCTCCCCACCAGCTGCTCTTTTCTTTTCTCTTTCTTTTCCCATCTTCAGTATATTCATCTTCCCATCCAAGAACCTTTATTTCCCCTAAGTAAGTACTTTGCTACATCCATACTCCATCCTTCCCATCCCTTATTCCTTTGAACCTTTCAGTTCGAGCTTTCCCACTTCATCGCAGCTTGACTAACAGCTACCCCGCTTGAGCAGACATCACCATGATTGAACAAGATGGATTGCACGCAGGTTCTCCGGCCGCTTGGGTGGAGAGGCTATTCGGCTATGACTGGGCACAACAGACAATCGGCTGCTCTGATGCCGCCGTGTTCCGGCTGTCAGCGCAGGGGCGCCCGGTTCTTTTTGTCAAGACCGACCTGTCCGGTGCCCTGAATGAACTGCAAGACGAGGCAGCGCGGCTATCGTGGCTGGCCACGACGGGCGTTCCTTGCGCAGCTGTGCTCGACGTTGTCACTGAAGCGGGAAGGGACTGGCTGCTATTGGGCGAAGTGCCGGGGCAGGATCTCCTGTCATCTCACCTTGCTCCTGCCGAGAAAGTATCCATCATGGCTGATGCAATGCGGCGGCTGCATACGCTTGATCCGGCTACCTGCCCATTCGACCACCAAGCGAAACATCGCATCGAGCGAGCACGTACTCGGATGGAAGCCGGTCTTGTCGATCAGGATGATCTGGACGAAGAGCATCAGGGGCTCGCGCCAGCCGAACTGTTCGCCAGGCTCAAGGCGAGCATGCCCGACGGCGAGGATCTCGTCGTGACCCATGGCGATGCCTGCTTGCCGAATATCATGGTGGAAAATGGCCGCTTTTCTGGATTCATCGACTGTGGCCGGCTGGGTGTGGCGGACCGCTATCAGGACATAGCGTTGGCTACCCGTGATATTGCTGAAGAGCTTGGCGGCGAATGGGCTGACCGCTTCCTCGTGCTTTACGGTATCGCCGCTCCCGATTCGCAGCGCATCGCCTTCTATCGCCTTCTTGACGAGTTCTTCTGAAGTAGATGCCGACCGGGATCCACTTAACGTTACTGAAATCATCAAACAGCTTGACGAATCTGGATATAAGATCGTTGGTGTCGATGTCAGCTCCGGAGTTGAGACAAATGGTGTTCAGGATCTCGATAAGATACGTTCATTTGTCCAAGCAGCAAAGAGTGCCTTCTAGTGATTTAATAGCTCCATGTCAACAAGAATAAAACGCGTTTCGGGTTTACCTCTTCCAGATACAGCTCATCTGCAATGCATTAATGCATTGGACCTCGCAACCCTAGTACGCCCTTCAGGCTCCGGCGAAGCAGAAGAATAGCTTAGCAGAGTCTATTTTCATTTTCGGGAGACGAGATCAAGCAGATCAACGGTCGTCAAGAGACCTACGAGACTGAGGAATCCGCTCTTGGCTCCACGCGACTATATATTTGTCTCTAATTGTACTTTGACATGCTCCTCTTCTTTACTCTGATAGCTTGACTATGAAAATTCCGTCACCAGCCCCTGGGTTCGCAAAGATAATTGCACTGTTTCTTCCTTGAACTCTCAAGCCTACAGGACACACATTCATCGTAGGTATAAACCTCGAAAATCATTCCTACTAAGATGGGTATACAATAGTAACCATGGTTGCCTAGTGAATGCTCCGTAACACCCAATACGCCGGCCGAAACTTTTTTACAACTCTCCTATGAGTCGTTTACCCAGAATGCACAGGTACACTTGTTTAGAGG |
| Zeocin | TGACTTGAGCCTAAAATCCGCCGCCTCCACCATTTGTAGAAAAATGTGACGAACTCGTGAGCTCTGTACAGTGACCGGTGACTCTTTCTGGCATGCGGAGAGACGGACGGACGCAGAGAGAAGGGCTGAGTAATAAGCGCCACTGCGCCAGACAGCTCTGGCGGCTCTGAGGTGCAGTGGATGATTATTAATCCGGGACCGGCCGCCCCTCCGCCCCGAAGTGGAAAGGCTGGTGTGCCCCTCGTTGACCAAGAATCTATTGCATCATCGGAGAATATGGAGCTTCATCGAATCACCGGCAGTAAGCGAAGGAGAATGTGAAGCCAGGGGTGTATAGCCGTCGGCGAAATAGCATGCCATTAACCTAGGTACAGAAGTCCAATTGCTTCCGATCTGGTAAAAGATTCACGAGATAGTACCTTCTCCGAAGTAGGTAGAGCGAGTACCCGGCGCGTAAGCTCCCTAATTGGCCCATCCGGCATCTGTAGGGCGTCCAAATATCGTGCCTCTCCTGCTTTGCCCGGTGTATGAAACCGGAAAGGCCGCTCAGGAGCTGGCCAGCGGCGCAGACCGGGAACACAAGCTGGCAGTCGACCCATCCGGTGCTCTGCACTCGACCTGCTGAGGTCCCTCAGTCCCTGGTAGGCAGCTTTGCCCCGTCTGTCCGCCCGGTGTGTCGGCGGGGTTGACAAGGTCGTTGCGTCAGTCCAACATTTGTTGCCATATTTTCCTGCTCTCCCCACCAGCTGCTCTTTTCTTTTCTCTTTCTTTTCCCATCTTCAGTATATTCATCTTCCCATCCAAGAACCTTTATTTCCCCTAAGTAAGTACTTTGCTACATCCATACTCCATCCTTCCCATCCCTTATTCCTTTGAACCTTTCAGTTCGAGCTTTCCCACTTCATCGCAGCTTGACTAACAGCTACCCCGCTTGAGCAGACATCACCATGGCCAAGTTGACCAGTGCCGTTCCGGTGCTCACCGCGCGCGACGTCGCCGGAGCGGTCGAGTTCTGGACCGACCGGCTCGGGTTCTCCCGGGACTTCGTGGAGGACGACTTCGCCGGTGTGGTCCGGGACGACGTGACCCTGTTCATCAGCGCGGTCCAGGACCAGGTGGTGCCGGACAACACCCTGGCCTGGGTGTGGGTGCGCGGCCTGGACGAGCTGTACGCCGAGTGGTCGGAGGTCGTGTCCACGAACTTCCGGGACGCCTCCGGGCCGGCCATGACCGAGATCGGCGAGCAGCCGTGGGGGCGGGAGTTCGCCCTGCGCGACCCGGCCGGCAACTGCGTGCACTTCGTGGCCGAGGAGCAGGACTGACCGACGCCGACCAACACCGCCGGTCCGACGCGGCCCGACGGGTCCGAGGCCTCGGAGATCCGTCCCCCTTTTCCTTTGTCGATATCATGTAATTAGTTATGTCACGCTTACATTCACGCCCTCCCCCCACATCCGCTCTAACCGAAAAGGAAGGAGTTAGACAACCTGAAGTCTAGGTCCCTATTTATTTTTTTATAGTTATGTTAGTATTAAGAACGTTATTTATATTTCAAATTTTTCTTTTTTTTCTGTACAGACGCGTGTACGCATGTAACATTATACTGAAAACCTTGCTTGAGAAGGTTTTGGGACGCTCGAAGGCTTTAATTTGCAAGCT |

**Figure S1.** Effect of temperature on cellobiose and cellobionate production by strain TH11 grown on 2% NaOH-pretreated wheat straw. Fermentations were performed at 43 and 45 °C. (a) cellobiose concentration and (b) cellobionate concentration. Data represent mean ± SD of triplicate cultures.
